# Medical 3D printing with polyjet technology: effect of material type and printing orientation on printability, surface structure and cytotoxicity

**DOI:** 10.1186/s41205-023-00190-y

**Published:** 2023-09-28

**Authors:** Karl H. Schneider, Gunpreet Oberoi, Ewald Unger, Klara Janjic, Sabrina Rohringer, Stefan Heber, Hermann Agis, Andreas Schedle, Herbert Kiss, Bruno K. Podesser, Reinhard Windhager, Stefan Toegel, Francesco Moscato

**Affiliations:** 1 Center for Biomedical Research and Translational Surgery, Medical University of Vienna, Waehringer Guertel 18-20, 1090 Vienna, Austria; 2 Ludwig Boltzmann Institute for Cardiovascular Research, Vienna, Austria; 3 Austrian Cluster for Tissue Regeneration, Vienna, Austria; 4 Center for Medical Physics and Biomedical Engineering, Medical University of Vienna, Waehringer Guertel 18-20, 1090 Vienna, Austria; 5 Austrian Center for Medical Innovation and Technology (ACMIT), Wiener Neustadt, Austria; 6 University Clinic of Dentistry, Medical University of Vienna, Sensengasse 2a, 1090 Vienna, Austria; 7 Institute of Physiology, Center for Physiology and Pharmacology, Medical University of Vienna, Schwarzspanierstraße 17, 1090 Vienna, Austria; 8 Department of Obstetrics and Gynecology, Division of Obstetrics and Feto-Maternal Medicine, Medical University of Vienna, Waehringer Guertel 18-20, 1090 Vienna, Austria; 9 Department of Orthopedics and Trauma Surgery, Karl Chiari Lab for Orthopaedic Biology, Medical University of Vienna, Waehringer Guertel 18-20, 1090 Vienna, Austria; 10 Ludwig Boltzmann Institute for Arthritis and Rehabilitation, Vienna, Austria

**Keywords:** Additive manufacturing, Inkjet, Medical 3D printing, Cytotoxicity

## Abstract

Due to its high printing resolution and ability to print multiple materials simultaneously, inkjet technology has found wide application in medicine. However, the biological safety of 3D-printed objects is not always guaranteed due to residues of uncured resins or support materials and must therefore be verified. The aim of this study was to evaluate the quality of standard assessment methods for determining the quality and properties of polyjet-printed scaffolds in terms of their dimensional accuracy, surface topography, and cytotoxic potential.

Standardized 3D-printed samples were produced in two printing orientations (horizontal or vertical). Printing accuracy and surface roughness was assessed by size measurements, VR-5200 3D optical profilometer dimensional analysis, and scanning electron microscopy. Cytotoxicity tests were performed with a representative cell line (L929) in a comparative laboratory study. Individual experiments were performed with primary cells from clinically relevant tissues and with a Toxdent cytotoxicity assay.

Dimensional measurements of printed discs indicated high print accuracy and reproducibility. Print accuracy was highest when specimens were printed in horizontal direction. In all cytotoxicity tests, the estimated mean cell viability was well above 70% (*p* < 0.0001) regardless of material and printing direction, confirming the low cytotoxicity of the final 3D-printed objects.

**Graphical Abstract:**

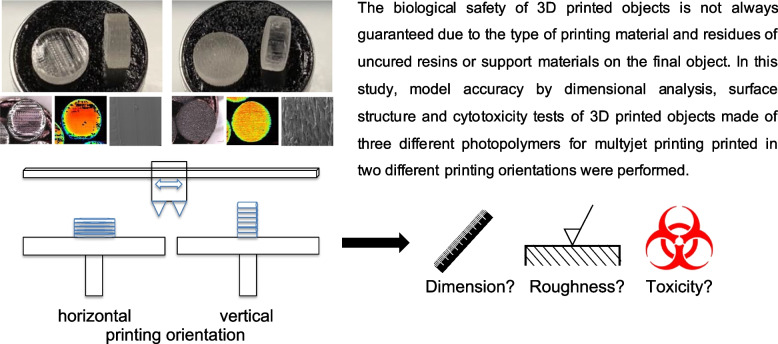

**Supplementary Information:**

The online version contains supplementary material available at 10.1186/s41205-023-00190-y.

## Introduction

Additive manufacturing from light-polymerized resins is increasingly employed in medicine due to its superior print quality and speed, opening up new possibilities in therapy [1]. As understanding of this technology grows, so does the demand for biologically safe materials for medical device additive manufacturing [2]. In a variety of surgical specialties, such as dentistry [3] and orthopedics [4], there are already numerous applications for 3D-printed objects due to their specialized customization capabilities. These 3D objects can be both off-the-shelf and highly personalized anatomic models, surgical guides or preoperative simulations [5–8], biodegradable screws [9], or functional prostheses [10].

Regarding printing with light polymerization, stereolithography (SLA) can be considered the pioneer among these technologies and is therefore still the most widely used. However, SLA printers operate at a lower print resolution than Multi Jet Manufacturing printers. For example, PolyJet™ printers from Stratasys (Eden Prairie, MN, United States of America) can reach 42.3 μm in the XY plane, representing one of the most accurate printing technologies [11, 12]. High-resolution objects with different module thicknesses in layers as thin as 16 µm and a Z resolution of 0.025 mm can be 3D-printed with high dimensional accuracy [13, 14]. Therefore, PolyJet™ printers are ideal for rapid prototyping of medical devices or surgical models [15]. However, the advancing clinical application of any printing technology relies on the availability of biocompatible printing materials with good printability [16–19]. Therefore, 3D-printed objects must be tested for toxic effects on clinically relevant cells in vitro prior to a possible clinical translation [20, 21].

It is well known that uncured liquid printing materials are cytotoxic. They are classified as causing severe eye irritation and may provoke an allergic skin reaction after repeated exposure. Only after the curing process the materials are non-toxic in their solid state [22]. Hence, all uncured residues must be removed during the cleaning process. With different print orientations, uncured material may become trapped in nascent structural compartments, leading to cytotoxicity during final application [23]. To address this issue, we included two groups of discs printed either in horizontal (XY) or vertical print (ZX) orientation, as the associated differences in the processes might result in different curing efficiencies. Therefore, we evaluated the printability and cytotoxicity of three different photocurable resins for medical applications, printed with two different printing orientations.

Since polyjet technology carries with it a particular dependence of the highly engineered equipment, one is often dependent on the materials of the equipment manufacturers. In this study, we wanted to start by looking at the evaluation process in general. Therefore, only materials from the manufacturer were compared. We examined three photopolymers proposed for use in PolyJet™ medical 3D printing: Vero Clear RGD810 (VC), Vero PureWhite RGD837 (VPW), and MED610 (M610). VC is a rigid transparent material designed to simulate PMMA (polymethyl methacrylate – acrylic). Its transparency makes VC ideal for investigating flow characteristics in accurate vascular models [24]. VPW has the same properties as VC regarding rigidity and structural stability but is white and opaque (Stratasys). Med610 is a transparent material for PolyJet systems from Stratasys. It represents the only one of the three tested materials specified as biocompatible and particularly suitable for medical applications in additive manufacturing due to its good dimensional stability.

This study was performed as a comparative laboratory study using an MTT cytotoxicity assay according to ISO guideline 10,993–5:2009 [25] by three independent laboratories of the Medical University of Vienna that are partners of the Austrian 3D printing consortium “Additive Manufacturing for Medical Research” (www.m3dres.org), namely the Center for Biomedical Research (CBMR), the Karl Chiari Laboratory for Orthopedic Biology at the Department of Orthopedics and Trauma Surgery (OTS), and the University Clinic of Dentistry (CDENT).

A detailed statistical analysis of the data obtained in this study was used not only to evaluate the short-term cytotoxicity of 3D-printed materials in their final stage but also to evaluate the quality of the test procedure and the influence of other factors such as the facility where the test was performed, the print orientation and the cell type used in the analysis.

## Materials and methods

### Tested Polyjet™ materials

All three printing materials (VC, VPW, M610) were purchased from Stratasys (Stratasys, Eden Prairie, MN, United States of America) and used according to the manufacturer’s guidelines. Mechanical properties were tested by our group before [26].

### Sample disc preparation

Discs were designed by a standardized geometry to fulfill dimensions according to the ISO guideline with a diameter of 5 mm and a height of 2 mm of VC, VPW, or M610 were printed in standard digital mode with a layer thickness of 30 µm (600 dpi) using PolyJet™ Connex3 Objet500 (Stratasys, Eden Prairie, MN, United States of America). The so-called light mode with matte printing function was used to provide a full enclosure of the model with the support material (SUP706). One set of discs was printed in vertical orientation (ZX), while the other was printed in horizontal orientation (XY). To prevent the presence of the acrylic support material (SUP706) as a remnant after printing, the crafted discs were cleaned strictly following the manufacturer’s (Stratasys) instructions including mechanical removal using water pressure and rinsing in 2% caustic soda (NaOH), 1% sodium metasilicate (Na2SiO3), and analytical grade isopropyl alcohol. The samples were dried overnight, resulting in better handling properties. Sterilization was then performed using 6% ethylene oxide / 94% carbon dioxide (GHC Gerling, Holz & Co). Disc dimensions were determined from 50 samples of each group using a Mitutoyo 7309 Pocket Thickness Gauge (0-10 mm range, 0.01 mm graduation, 20-µm accuracy).

### Surface topography analysis

Two different imaging techniques were used for surface topography analysis. Scanning electron microscopy (SEM) was used to evaluate the surface topography at high resolution. For SEM imaging, the 3D-printed parts were placed on metal holders in two different orientations to allow viewing of the long and short edges of the disk-shaped samples. The samples were sputter-coated with gold at 20 mA for 120 s and analyzed with a Zeiss EVO 10 (Zeiss, Austria). Additionally, roughness profiles and disc dimensions were determined by performing one-shot high-resolution 3D microscopic measurements with a resolution of 0.1 μm using a VR-5200 3D optical profilometer (Keyence, Keyence International, Mechelen, Belgium). This device acquires measurement data at 800,000 points in four seconds with a working distance of 75 mm and offers a resolution of 0.1 μm. 

### Clinical specimens

In total, 6 clinical specimens from 6 osteoarthritis (OA) patients (cartilage: *n* = 3 patients; synovium: *n* = 3 patients) were obtained from the ViBiMeD biobank of the Department of Orthopedics and Trauma Surgery (vote no.: 1822/2017). This biobank includes clinical waste tissue (e.g., cartilage, synovium) from patients undergoing surgery at the Department. All patients have donated their waste tissues for scientific research with written informed consent. The ethics committee of the Medical University of Vienna (vote no.: 2132/2019) approved the use of the specimens for the present study. Placental tissues for primary endothelial cell isolation were obtained from the Department of Obstetrics and Gynecology, Medical University of Vienna (vote no:1602/2018). All clinical samples were used in a pseudonymized manner. No patient names, images, or data of any kind that would allow the identification of individual patients are published or made public.

### Primary chondrocytes and synovial fibroblasts

After excision, the clinical tissues were immediately put in a sterile tube at + 4°C and carefully washed with phosphate-buffered saline (PBS) to reduce the number of blood cells. Fibroblast-like synoviocytes (FLS) were isolated from the synovial tissue by placing tissue fragments in culture flasks, allowing the outgrowth of cells. After two days, cells were washed with PBS, and the medium was changed. After 10 to 14 days, the tissue fragments were separated from the culture. Primary chondrocytes were isolated from cartilage tissue of the tibia plateau and femoral condyles. First, cartilage tissue was cut into small pieces and incubated with collagenase B overnight. During this time, the bottle was shaken permanently, and a temperature of 37°C was maintained. After incubation, the mixture was filtered to eliminate any undigested cartilage particles. Then, the cell suspension was centrifuged before washing the cells with PBS and resuspending them with the medium. FLS and primary chondrocytes were maintained in Dulbecco’s modified Eagle’s medium (DMEM; Gibco, Life Technologies, Carlsbad, CA), supplemented with 10% fetal bovine serum, 1% penicillin–streptomycin, and 0.1% Amphotericin B. Cultivation took place in 75 cm^2^ culture flasks in a humidified atmosphere with 5% CO_2_ at 37°C. The medium was changed 2–3 times a week. Chondrocyte cultures were only used in P0 to prevent the loss of the specific chondrocyte phenotype (7). Passaging of FLS was performed by incubating the cell monolayers for 3 min with 3 ml trypsin–EDTA preheated to 37°C. FLS were passaged at 90% confluence and used for experiments in passages 4 (P4) and P5 (6). To determine cell numbers for seeding, cells were counted using the trypan blue method and the Neubauer counting chamber.

### Primary endothelial cells from human umbilical cord vein (HUVEC)

Primary endothelial cells were isolated according to an adapted protocol described before [27]. Briefly, the placental vascular tree was rinsed with phosphate-buffered saline (PBS) supplemented with heparin (50 IU/ml) and antibiotics (1% penicillin/streptomycin). The umbilical cord vein was filled with collagenase I solution (0.2% w/v) (Roche, cat. no. 103586) using a 21 G needle, clamped on both sides, and incubated in a pre-warmed sterile PBS bath for 15 min. Afterwards, the collagenase I solution was collected with detached HUVECs in a 50 ml centrifugation tube. The collected cell suspension was centrifuged with 300 × g for 5 min. The resulting cell pellet was resuspended in media supplemented with 10% FCS and plated onto a T25 cell culture flask. Once the flask reached confluency, cells were split into a T75 flask to start with the standard cell culture routine.

### L929 cell line

The L929 cell line was purchased commercially (NCTC clone 929, Lcell, L929, derivative of Strain L, ATCC® CCL-1™, American Type Culture Collection, Manassas, VA, USA). It represents a murine cell line from normal subcutaneous areolar and adipose tissue of a 100-day-old male C3H/An mouse. Cells were cultivated in Dulbecco’s modified Eagle’s medium (DMEM; Gibco, Life Technologies, Carlsbad, CA), supplemented with 10% fetal bovine serum and 1% penicillin–streptomycin. When necessary, cells were cryopreserved at -196°C in 10% DMSO in full culture medium and thawed 14 days before experiments. After thawing and quickly resuspending with medium, cells were centrifuged with a following change of medium to minimize the concentration of DMSO.

### Cytotoxicity tests

#### MTT assay

Three institutions performed an interlaboratory test according to ISO guidelines using the MTT assay with the L929 cell line. Briefly, L-929 cells were seeded at a concentration of 6 × 10^5^ cells/ml into 24 well plates. One day after seeding, the medium was changed to remove dead cells. Then, the cells were overlaid with the specimens which covered 10% of the surface area to be exposed overnight. Non-toxic glass discs of the same size as the 3D-printed discs were used as a negative control, whereas discs of cytotoxic carboxylate cement were used as positive control. After 24 h, the specimens were removed, and the medium was replaced with MTT in medium at 1 mg/ml. After 2 h of incubation, the MTT solution was removed, and the resulting crystals were solubilized with DMSO. The solution was transferred into a 96-well plate, and the absorbance was measured at 570 nm with a reference wavelength of 650 nm. The viability of cells exposed to the printed test materials was compared across the test and control groups. The viability of cells is expressed as percent relative to the negative control set at 100%. The L929 cell line was used in all laboratories, while experiments with the primary cells were conducted with the respective cells only in one laboratory: FLS and chondrocytes at OTS, and HUVECs at CBMR.

#### Toxdent test

The Toxdent test was performed at CDENT as previously described [28]. Briefly, 3 × 10^4^ L929 cells per ml were seeded onto 6-well culture plates and were exposed to the specimens for 72 h. Then, cells were trypsinized and counted in a suspension of 500 µl over 30 s using flow cytometry (FACSCalibur, Becton, Dickinson). The data are presented as fold change relative to untreated cells.

### Statistical data analysis

#### Applicable to all analysis

Statistical analyses were performed using IBM SPSS Statistics 28.0.1.0. No adjustment for multiplicity was performed due to the exploratory character of this study, and the results need to be interpreted accordingly. Approximate normal distribution of residuals was checked visually, where indicated data were log_10_ transformed before analysis to stabilize the residual distribution. Only two-sided *p*-values are reported, and *p*-values ≤ 0.05 were considered statistically significant. For a more detailed explanation of the statistical considerations in this study, see the Supplementary Methods Sect. 1.

#### Surface topography data processing

For dimension measurements, two separate 2-way analyses of variance were performed to investigate whether the precision in height or diameter, defined as the absolute deviation from the target value (height 2 mm, diameter 5 mm), differs between materials and whether this in turn is affected by printing direction.

Two parameters reflecting roughness (mean peak, Ra, and averages roughness, Rz) were analyzed separately by mixed linear models. There were 4 specimens per material (VC, VPW, M610) per printing direction (horizontal, vertical), i.e., 24 in total. Each specimen was assessed three times for Ra and three times for Rz, Significant ‘material*direction’ interaction terms were broken down using contrasts comparing the two printing directions within each material.

#### MTT multi-laboratory experiment

The research question regarding the MTT assay was whether the cytotoxicity of materials depends on printing direction and whether this depends on the laboratory, corresponding to a three-way interaction hypothesis. The factors were 1) ‘material’ 2) ‘printing direction’ with the levels “vertical”, “horizontal” and “not printed”, whereby the materials “glass” and “cement” were always not printed and 3) ‘laboratory’ with the levels “CBMR”, “CDENT” and “OTS”. A linear mixed model was applied for statistical analysis, including the three above-mentioned factors as fixed factors. All values corresponding to “glass” were not included in the statistical model, as they were used for normalization. From the resulting model, least square means were calculated for each condition with their 95% confidence intervals and tested against the a priori-defined cut-off for toxicity of 70% viability.

#### MTT primary cell experiment

Primary cells were exposed to the materials to test potential toxicity in two laboratories. The CBMR laboratory analyzed HUVECs from 5 different donors, whereas the OTS analyzed both chondrocytes and FLS from three donors, whereby each of the three patients donated both chondrocytes and FLS. Cells of each donor were exposed to glass, cement, M610, VC, and VPW, the latter three of which in both printing directions, i.e., 8 conditions. The analysis was performed by a linear mixed model. The fixed factors were 1) ‘material’ 2) ‘printing direction’ with the levels “vertical”, “horizontal” and “not printed”, whereby “cement” was always not printed and 3) ‘cell type with the levels “HUVECs”, “chondrocytes” and “FLS”. The donors were included as levels of a random factor. To stabilize the residual distribution, the normalized values were log_10_ transformed before analysis. The estimated means (of log_10_ transformed values) for each experimental group of interest were tested against 1.8451, i.e. against 70% on the original scale, to assess whether the percentage viability differs from this value.

#### Toxdent

To test whether materials are cytotoxic as measured by the Toxdent-Assay, a linear mixed model was used. Samples were prepared in 6-well plates, of which 60 were used, corresponding to 360 data points. Plates were randomly assigned to each of the 5 experimental conditions/materials (neg CTR, pos CTR, VC, VPW, M610), i.e., each plate contained only wells with a specific material in a certain printing direction. To allow an intuitive interpretation of the results (i.e., in % viability), data were normalized to the mean of the neg CTR values, separately by experimental run. The printing direction was used as a binary fixed factor, and the materials were represented as a fixed factor with four levels, since the neg CTR was omitted from the analysis as it was used for normalization. Before analysis, the data were log_10_-transformed; therefore, least square means are geometric means. Each of the least-square means was tested against 70% to assess whether the geometric mean is different from this priori defined threshold for toxicity.

## Results

### Surface topography

**Fig. 1:**
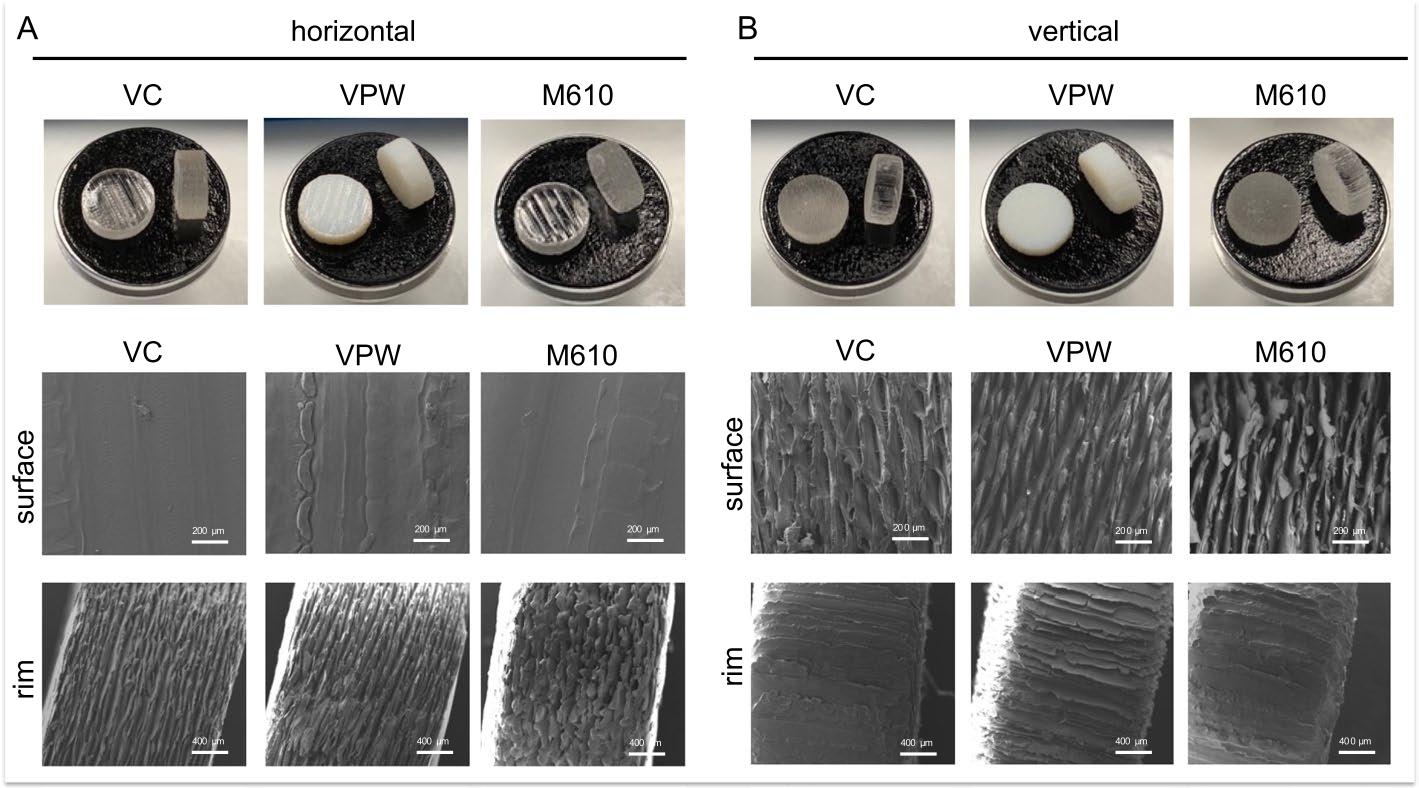
Sample discs were 3D-printed from three different materials Vero Clear (VC), Vero Pure White (VPW), and Med610 (M610), using either (**A**) horizontal or (**B**) vertical printing orientation. Macroscopic images and scanning electron microscopy showed considerable differences in the structure of the disc surfaces and rims

According to the results from scanning electron microscopy, all three materials showed similar surface structures under the same printing orientation condition (Fig. 1). Horizontal printing resulted in smooth surfaces in the upper and lower areas and in fine grooves on the surface along the circumference of the cylindrical disc. Vertical printing resulted in fine grooves in the upper and lower areas and along the rims oriented orthogonally to the long axis of the cylindrical disc.

### Roughness and disc dimensions

**Fig. 2:**
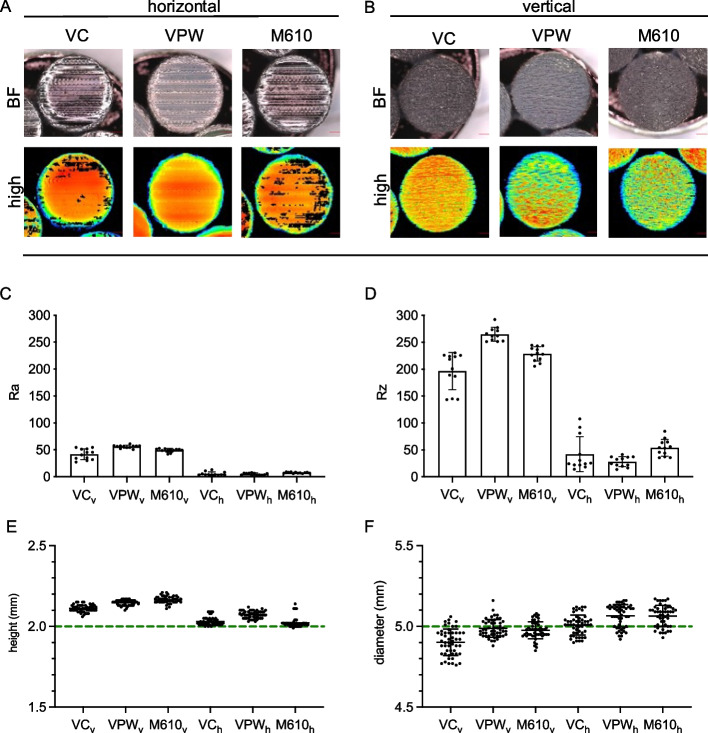
(**A**,**B**) Surface scans using a high-resolution 3D macroscopic measurement system (VR-5200 3D optical Profilometer, 0.1 µm resolution) using bright field (real) and high-resolution laser (high). High-resolution images allowed to determine values for surface roughness in (**C**) mean peak (Ra) and (**D**) averaged roughness (Rz). Every dot represents the arithmetic mean of three technical replicates. (**E**, **F**) Dimensional print accuracy was determined by manually measuring height and diameter discs from each group (*n* = 50 specimens, one measurement/specimen) using a Mitutoyo 7309 Pocket Thickness Gauge. Values where related to STL file dimensions (dashed line). Small subscript v refers to samples printed in vertical direction, subscript h in horizontal direction

During high-resolution 3D micro measurements, it was observed that transparent materials exhibited gaps in the measurement signals, as seen in samples printed with VC and M610 with horizontal print orientation (Fig. 2A, black spots). Opaque samples offered sufficient reflection to perform a gap-free evaluation with the line scanner due to the material itself or higher surface roughness (Fig. 2B). Despite the effect of reflection, a sufficient number of positions on all samples could be used for the measurement. For all three materials, horizontal printing resulted in much lower roughness based on mean peak (Ra) when the materials were printed horizontally compared to vertically (*p* < 10^–8^ each). This was significantly dependent on the material (*p* = 0.021), with the most prominent effect of printing direction for the material VPW (Fig. 2C). The analogous statistical analysis with averaged roughness (Rz) as target variable showed similar results (vertical vs. horizontal *p* < 10^–7^ for each material, material-dependent difference *p* = 0.059, Fig. 2D).

Manual dimensional measurements of printed discs indicated high print accuracy and reproducibility. The digital model was designed to print discs of 2 mm height and 5 mm diameter. Regarding height, statistical analysis showed that the absolute deviation from the 2 mm target was much lower, indicating a better precision when materials were printed horizontally compared to vertically (*p* < 10^–39^ for each material). However, these differences were not equal for each material (*p* = 8 × 10^–23^) and were most pronounced for M610 (Fig. 2E). Concerning diameter, however, the material-dependent effect of printing direction on precision was more pronounced and statistically significant (*p* = 6 × 10^–12^). M610 showed a slightly better precision when printed vertically compared to horizontally (*p* = 0.004). This was also the case for VPW (*p* < 0.001). Conversely, VC resulted in a better precision when printed horizontally (*p* < 0.001). Notably, while statistically significant, the deviations from the target diameter were low for all printing directions and materials and thus considered irrelevant (Fig. 2F).

### Cytotoxicity assays show cytocompatibility

**Fig. 3:**
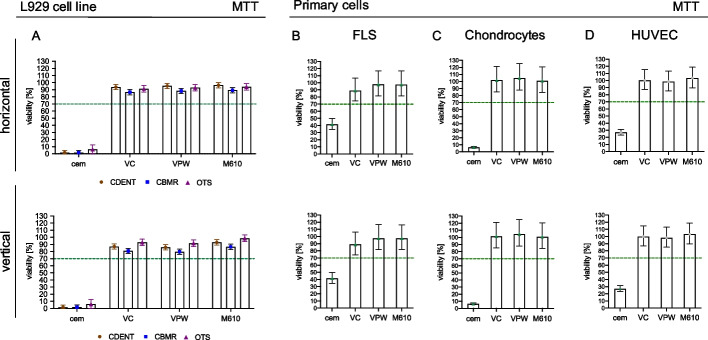
(**A**) Comparison of MTT cytotoxicity test results from three different laboratories (CBMR, OTS, and CDENT) using the L929 cell line and primary cells isolated from three different human tissues, (**B**) fibroblast-like synoviocytes (FLS) (*n* = 3 patients), (**C**) human chondrocytes (*n* = 3 patients), and (**D**) human umbilical vein endothelial cells (HUVEC) (*n* = 4 patients). All data are expressed as percent viability (least square arithmetic means ± the 95% confidence intervals), relative to values in the presence of the negative control (glass, 100% viability). Cytotoxic carboxylate cement (cem) was used as positive control. Values above 70% viability (dashed line) were considered to confirm the absence of cytotoxic effects of the tested sample. Of note, 95% CI error bars that do not cross the dotted horizontal 70% line are significantly (*p* < 0.05) different from 70%

**Fig. 4:**
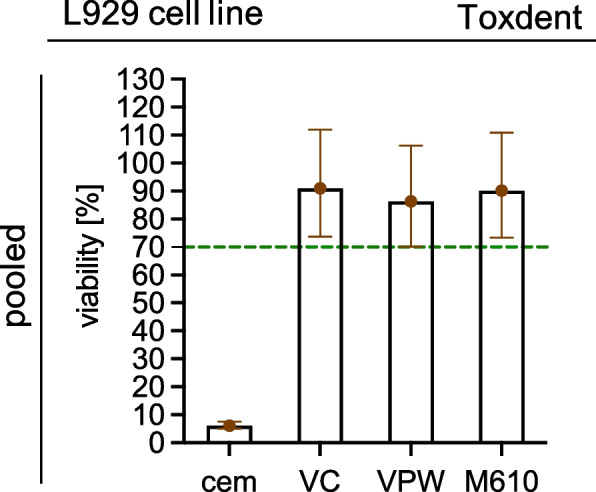
Toxdent performed using L929 cell line. All data are expressed as percent viability (least squares arithmetic means ± 95% confidence intervals), relative to values in the presence of the negative control (glass, 100% viability). Cytotoxic carboxylate cement (cem) was used as positive control, values above 70% viability (dashed line) were considered to confirm the absence of cytotoxic effects of the sample. Those 95% confidence intervals which do not cross the dotted line indicate significantly (*p* < 0.05) different mean values from 70%. Importantly, it was justified to also statistically estimate pooled mean values for horizontal and vertical samples, as there was no evidence for an effect of printing direction (*p* = 0.84)

Cytotoxicity tests were performed according to ISO norm 10,993–5:2009 using the MTT assay. Values above 70% viability were considered to indicate the absence of cytotoxic effects of the tested material. Individual laboratory results obtained with L929 cells were analyzed to evaluate the reproducibility of experiments performed in different laboratories by different operators. Results of all three laboratories supported the same conclusion, namely that none of the materials had cytotoxic effects, irrespective of their printing direction. In particular, the statistical analysis showed that the estimated mean viability of L929 cells was significantly above 70% (*p* < 0.0001), irrespective of the tested material, the printing direction, and the laboratory. Furthermore, our results show that the positive control was effective in consistently causing severe cytotoxicity (Figs. 3 and 4).

Besides the ring trial, primary cells were tested according to the same protocol. FLS, chondrocytes, and HUVEC primary cells showed no cytotoxic effect in any tested preparation (Fig. 3). The viability of chondrocytes, FLS, and HUVECs was significantly higher than 70% after exposure to M610, VC, and VPW in both printing directions (*p* < 0.015 each), confirming that none of the materials was cytotoxic, irrespective of how it was printed. The cement material (i.e., the positive control), however, significantly impaired the cell viability (*p* < 0.0001), with chondrocytes being most susceptible to cement, followed by HUVECs and FLS. Despite these differences, the positive controls were successful insofar as the viability of all cell types was significantly below 70% (*p* < 0.0001 each).

In line with the ring trial’s results, the analysis of the Toxdent showed neither cytotoxicity for the materials nor any effect of the printing direction (material*printing direction interaction *P* = 0.93, main effect of printing direction 0.84). The estimated mean viability of L929 cells exposed to one of the three materials of interest, pooled for both printing directions, was significantly above 70% (M610 *P* = 0.019, VC *P* = 0.015, VPW *P* = 0.0496). In contrast, the estimated viability was below 70% in the positive control (*p* < 0.0001). To improve clarity, estimated viability was plotted separately by printing direction (Fig. 4, pooled estimates plotted in Online Supplement).

## Discussion

The multi jet manufacturing technology considered in this work can be utilized in tissue engineering to build complex scaffolds as testing platforms and medical devices. The chemical composition of the printing and carrier materials is mainly based on Poly(methyl methacrylate) (PMMA) or polyethylene glycol (PEG), which are known to cause specific toxic effects upon exposure to cells, tissues, and organisms. This toxicity is lost after the complete polymerization of the materials. However, the potential leaching of unreacted monomers of the photoinitiators and residual support material from the finished 3D object into the environment could cause biocompatibility issues [29]. Therefore, the printing process is always followed by intensive washing steps to completely remove the carrier material from the 3D-printed objects, and it is recommended to thoroughly test these objects for cytotoxicity to evaluate safety.

This study investigated the printability and cytotoxicity of three different PolyJet™ printing materials suitable for biological contact. In addition, the changes in surface topography and the potential cytotoxic effect of the possible retention of toxic unpolymerized photoinitiators or support material were determined. Considering the cytotoxic effect of these materials without additional post-processing steps other than final cleaning, MTT and Toxdent tests were performed. With our selection of materials, it was intended to illustrate a possible difference between materials tested by the manufacturer as biocompatible and those without such a certificate. The photosensitive resins used for 3D printing are considered highly allergenic and sometimes toxic in their liquid state. Only after complete polymerization and final purification do these materials become harmless on contact. This property was confirmed in the cytotoxicity studies we conducted for all materials tested. Interestingly, no significant difference in cytocompatibility was found between the materials classified as biocompatible and those not tested for that. Our results suggest that the chosen materials can be printed with horizontal or vertical printing orientation within the technical range of accuracy. All three tested materials showed no cytotoxic effects when printed.

From the material manufacturer, no impact of print orientation has been mentioned on printing accuracy; however, we noticed significant differences during the study. As such, components in horizontal orientation were printed particularly accurately with deviations below the measurement accuracy we applied. Vertically printed discs showed slightly higher deviations. This might be attributed to the fact that the layer-by-layer build-up in vertical orientation is done by a “coarser” system using a stepper motor for movements. In horizontal print orientation (XY), the build-up is primarily defined by the nozzle movement with a high motion resolution of 600 dpi without any additional offset. A nozzle dot is at 30 µm diameter (600 dpi), and thus, we have a deviation of + 1–2 dots. However, considering the small size of the sample discs, the deviations might still be regarded as relatively big; however, this would be reduced when printing larger parts.

The total surface area of a 3D-printed object plays a vital role in the print quality. As electron microscopy images showed, horizontal printing always produced a low surface profile in XY dimensions and a high profile in XZ. For vertical prints, the reverse situation was observed. An increased surface area or roughness of a surface means an increased risk of trapping residues of the support material or unpolymerized photoinitiators. Therefore, a possible influence of different print orientations on the cytocompatibility of 3D constructs was also investigated in this study. Several reports have documented the effect of print orientation on the supporting structures (SLA and FDM) / support material (PolyJet) required, print time, layering, polymerization, surface roughness, mechanical properties, and print accuracy [30–33]. A study investigating the influence of print orientation on polyethylene-based material with an FDM printer highlighted that all the mechanical and tribological properties were increased by 30–60% when printed in X-direction. Recently, the influence of printing orientation on the multi-material interface using multijet printing technology on the fracture strength was also investigated. Vertically printed specimens (XZ-plane) generally exhibited better fracture strength [34]. Regarding the accuracy of multijet printing alone, a study on the fabrication of mandibular full dentures showed that the highest accuracy was achieved at a build alignment of 45 °C [35].

In the present work, it can be seen as an advantage that the support material used (i.e., SUP706) is highly soluble in NaOH and, unlike other stiffer support materials (e.g., in extrusion printing), can be easily removed without affecting the dimensional accuracy of the samples. In the stereolithographic printing process using stiffer support materials, the support material needs to be removed mechanically or chemically, damaging the models’ surface. Cleaning is also crucial when using high-temperature support materials, as material dust is generated in the process.

M610 is the only candidate among the materials in this study that has already been approved for medical application according to standard ISO-10993 (Supplement Data). The manufacturer, Stratasys, warrants its biocompatibility at extended contact with skin (up to 30 days) and short-term contact with mucosal surfaces (up to 24 h). Furthermore, M610 is already in clinical use as a material for drilling and cutting guides in dental applications [36]. However, our experiments did not reveal increased cytotoxicity, even with VC and VPW materials, which are not yet approved for medical use. A recent study also found that even M610 had adverse effects on keratinocytes, suggesting that 3D printing materials may have cell-specific effects [37]. This observation explains the rationale for our study, in which we investigate the impact of polyjet-printed materials with potential for clinical use on primary cells explicitly exposed to these materials. This study holds therefore significant clinical value as it ventures beyond the conventional exploration of cytotoxicity in materials limited to the declared biocompatible ones.

Previously, the material M610 has been the primary choice because of its reported biocompatibility. However, as a wider range of polyjet materials with diverse mechanical and optical properties becomes available, the potential for advancements in biomedical applications has expanded. Herein lies the significance of this study, which proposes a systematic and standardized evaluation protocol for polyjet materials beyond M610. This protocol has the potential to broaden the scope of possibilities for fabricating components suitable for cell-culture studies, microfluidic setups, and even bone scaffolds [38–40]. By exploring a variety of materials, researchers and clinicians can now assess their compatibility with biological systems, paving the way for more advanced and tailored applications in medical research and practice.

Three independent laboratories used the same procedure to compare their results. Their results confirm that cytotoxicity tests according to the ISO standard provide reproducible and sensitive data. There was no evidence for different outcomes between laboratories, which further strengthens our conclusion that the tested materials do not exert cytotoxic effects. All tested materials, including M610, did not cause the investigated cells to exhibit viability values indicative of cytotoxicity. Importantly, not only the average viability percentages in each experimental condition were above 70%, but also their 95% confidence intervals. This indicates that our data are not compatible with mean values below 70% and thus do not suggest toxicity of the materials.

As the cleaning process is vital to preventing cytotoxicity, it can be concluded that in this setup the standard cleaning process recommended by the manufacturer was sufficient to remove unpolymerized resin and support material from the 3D-printed objects. However, longer washing steps with multiple cycles of acid and waterjet cleaning might be required for more complex structures in terms of geometry. Literature shows also that cleaning process using sonication can even optimize biocompatibility in-vivo and should be therefore considered [41].

A limitation of this study is that regardless of the reliable and consistent results obtained on cytotoxicity by using a cell line, the overall physiological significance of such cells is probably minor. Cell lines may not possess the critical morphological or functional characteristics of primary cells in vivo, making it challenging to translate the results into a specific clinical situation [42, 43]. Therefore, including primary cells or even 3D microtissues [44, 45] could provide more clinically relevant data on the interactions between cells and 3D-printed biomaterials.

Here, we investigated the cytotoxic effects of 3D-printed objects on three primary cell types such as chondrocytes, FLS, and endothelial cells. In the field of orthopedics, articular chondrocytes and cells of the synovial membrane are amongst the most prominent cells to interact with 3D-printed materials used for surgical implants, guides, or scaffold-based bioengineering approaches [46], thus providing the rationale for including them in our biocompatibility study. Fibroblasts are organ-specific cells highly involved in extracellular matrix synthesis of connective tissues [47], and endothelial cells provide the barrier between blood and tissue by lining the inner walls of our blood vessels [48]. Primary cells from up to four different donors for each cell type were used in this study.

Our results from primary cells confirmed the cytocompatibility of the tested specimens. However, in our setup, FLS and HUVEC were less affected by the toxic positive control than the L929 cell line or primary chondrocytes. This observation indicates that different primary cells may have variable sensitivities towards cytotoxic triggers, suggesting the need for cytotoxicity testing on a case-to-case basis. It might also argue in favor of including a standardized cell line as an internal control for cytotoxicity screening tests involving multiple laboratories.

## Conclusion and limitations

This report showed that the MTT and Toxdent tests are suitable for evaluating 3D-printed materials for their cytotoxic capacity with optimal reproducibility and comparability in results. Our experiments also showed that the print orientation did significantly alter surface properties of 3D-printed objects, but did not cause limitations in cell viability due to the possible leaching of residual uncured photoinitiators or the support material. However, this study did not investigate long-term incubation of materials or testing of modifications or custom-made resins, but provides a solid basis for future experiments in these directions. Our aim here was to take a first step in demonstrating the overlap in material testing between different clinical areas. The strengths of interdisciplinarity should be applied across disciplines especially when 3D printing applications are involved.

### Supplementary Information

**Additional file 1.**

## Data Availability

The raw/processed data required to reproduce these findings are attached to this manuscript as Supplementary files.

## References

[1] Khorsandi D (2021). 3D and 4D printing in dentistry and maxillofacial surgery: Printing techniques, materials, and applications. Acta Biomater.

[2] Guttridge C (2022). Biocompatible 3d printing resins for medical applications: a review of marketed intended use, biocompatibility certification, and post-processing guidance. Annals of 3D Printed Med.

[3] Oberoi G (2018). 3D Printing-Encompassing the Facets of Dentistry. Front Bioeng Biotechnol.

[4] Wixted CM (2021). Three-dimensional Printing in Orthopaedic Surgery: Current Applications and Future Developments. J Am Acad Orthop Surg Glob Res Rev..

[5] Ballard DH (2020). Medical 3D Printing Cost-Savings in Orthopedic and Maxillofacial Surgery: Cost Analysis of Operating Room Time Saved with 3D Printed Anatomic Models and Surgical Guides. Acad Radiol.

[6] Ibrahim D (2009). Dimensional error of selective laser sintering, three-dimensional printing and PolyJet models in the reproduction of mandibular anatomy. J Craniomaxillofac Surg.

[7] Hatamikia S (2020). Additively Manufactured Patient-Specific Anthropomorphic Thorax Phantom With Realistic Radiation Attenuation Properties. Front Bioeng Biotechnol.

[8] Oberoi G (2020). 3D Printed Biomimetic Rabbit Airway Simulation Model for Nasotracheal Intubation Training. Front Vet Sci.

[9] Dhandapani R (2020). Additive manufacturing of biodegradable porous orthopaedic screw. Bioact Mater.

[10] Li HZ (2019). Dental ceramic prostheses by stereolithography-based additive manufacturing: potentials and challenges. Adv Appl Ceram.

[11] Gear JI (2016). Abdo-Man: a 3D-printed anthropomorphic phantom for validating quantitative SIRT. EJNMMI Phys.

[12] Gear JI (2014). Development of patient-specific molecular imaging phantoms using a 3D printer. Med Phys.

[13] Osman RB (2017). 3D-printing zirconia implants; a dream or a reality? An in-vitro study evaluating the dimensional accuracy, surface topography and mechanical properties of printed zirconia implant and discs. J Mech Behav Biomed Mater.

[14] Lau I (2019). Quantitative and qualitative comparison of low- and high-cost 3D-printed heart models. Quant Imaging Med Surg.

[15] Petzold R, Zeilhofer HF, Kalender WA (1999). Rapid protyping technology in medicine–basics and applications. Comput Med Imaging Graph.

[16] Hoang D (2016). Surgical applications of three-dimensional printing: a review of the current literature & how to get started. Ann Transl Med.

[17] Ma Y (2019). Three-dimensional printing biotechnology for the regeneration of the tooth and tooth-supporting tissues. Biotechnol Bioeng.

[18] Maier J (2019). Imitating human soft tissue on basis of a dual-material 3D print using a support-filled metamaterial to provide bimanual haptic for a hand surgery training system. Quant Imaging Med Surg.

[19] Sta Agueda JRH, et al. 3D printing of biomedically relevant polymer materials and biocompatibility. MRS Commun. 2021;11(2):197–212. 10.1557/s43579-021-00038-8 PMC8075026 33936866

[20] Oberoi G (2021). The impact of 3D-printed LAY-FOMM 40 and LAY-FOMM 60 on L929 cells and human oral fibroblasts. Clin Oral Investig.

[21] Kurzmann C (2017). Evaluation of resins for stereolithographic 3D-printed surgical guides: the response of L929 Cells and human gingival fibroblasts. Biomed Res Int.

[22] Gonzalez G (2020). Materials Testing for the development of biocompatible devices through Vat-polymerization 3D printing. Nanomaterials (Basel).

[23] Bao YY, Paunovic N, Leroux JC (2022). Challenges and opportunities in 3D printing of biodegradable medical devices by emerging photopolymerization techniques. Ad Funct Mater.

[24] Aycock KI, Hariharan P, Craven BA (2017). Particle image velocimetry measurements in an anatomical vascular model fabricated using inkjet 3D printing. Exp Fluids.

[25] Oberoi G (2020). Titanium dioxide-based scanning powder can modulate cell activity of oral soft tissue - Insights from in vitro studies with L929 cells and periodontal fibroblasts. J Prosthodont Res.

[26] Königshofer M (2021). Mechanical and dimensional investigation of additive manufactured multimaterial parts. Front Phys.

[27] Crampton SP, Davis J, Hughes CC (2007). Isolation of human umbilical vein endothelial cells (HUVEC). J Vis Exp.

[28] Franz A (2014). Cytotoxicity of post and core composites as a function of environmental conditions. Dent Mater.

[29] Kim GT (2022). Cytotoxicity, colour stability and dimensional accuracy of 3D printing resin with three different photoinitiators. Polymers (Basel)..

[30] Navarro J (2019). Effect of print orientation on microstructural features and mechanical properties of 3D porous structures printed with continuous digital light processing. Rapid Prototyping J.

[31] Hanon MM, Alshammas Y, Zsidai L (2020). Effect of print orientation and bronze existence on tribological and mechanical properties of 3D-printed bronze/PLA composite. Int J Adv Manuf Technol.

[32] Domínguez-Rodríguez G, Ku-Herrera JJ, Hernández-Pérez A (2018). An assessment of the effect of printing orientation, density, and filler pattern on the compressive performance of 3D printed ABS structures by fuse deposition. Int J Adv Manuf Technol.

[33] Naik M, Thakur DG, Chandel S (2022). An insight into the effect of printing orientation on tensile strength of multi-infill pattern 3D printed specimen: Experimental study. Mater Today: Proc.

[34] Vu IQ (2018). Characterizing the effect of print orientation on interface integrity of multi-material jetting additive manufacturing. Addit Manuf.

[35] Gao H (2021). The effect of build orientation on the dimensional accuracy of 3D-printed mandibular complete dentures manufactured with a multijet 3D printer. J Prosthodont.

[36] Torok G (2020). Effects of disinfection and sterilization on the dimensional changes and mechanical properties of 3D printed surgical guides for implant therapy - pilot study. BMC Oral Health.

[37] Schmelzer E (2016). Response of primary human bone marrow mesenchymal stromal cells and dermal keratinocytes to thermal printer materials in vitro. J Med Biol Eng.

[38] Mustahsan VM (2021). Biocompatible customized 3D bone scaffolds treated with CRFP, an osteogenic peptide. Bioengineering (Basel).

[39] Cenhrang K (2022). 3D printed devices with integrated collagen scaffolds for cell culture studies including transepithelial/transendothelial electrical resistance (TEER) measurements. Anal Chim Acta.

[40] Currens ER (2022). Evaluation and optimization of PolyJet 3D-printed materials for cell culture studies. Anal Bioanal Chem.

[41] Ngan CGY (2019). Optimising the biocompatibility of 3D printed photopolymer constructs in vitro and in vivo. Biomed Mater.

[42] Pastor DM (2010). Primary cell lines: false representation or model system? a comparison of four human colorectal tumors and their coordinately established cell lines. Int J Clin Exp Med.

[43] Kaur G, Dufour JM (2012). Cell lines: Valuable tools or useless artifacts. Spermatogenesis.

[44] Oberoi G (2018). Contraction dynamics of rod microtissues of gingiva-derived and periodontal ligament-derived cells. Front Physiol.

[45] Oberoi G (2020). Contraction dynamics of dental pulp cell rod microtissues. Clin Oral Investig.

[46] Shie MY (2017). 3D Printing of Cytocompatible Water-Based Light-Cured Polyurethane with Hyaluronic Acid for Cartilage Tissue Engineering Applications. Mater (Basel)..

[47] Plikus MV (2021). Fibroblasts: origins, definitions, and functions in health and disease. Cell.

[48] Kruger-Genge A (2019). Vascular endothelial cell biology: an update. Int J Mol Sci.

