# Supplementary material for: Medical 3D printing with polyjet technology: effect of material type and printing orientation on printability, surface structure and cytotoxicity
Source: 3D Print Med. 2023 Sep 28;9:27. doi: 10.1186/s41205-023-00190-y (PMC10540425; doi:10.1186/s41205-023-00190-y)
Supplement: Supplementary file 1 — Additional file 1. [file 41205_2023_190_MOESM1_ESM.pdf]

Supplemental Methods:

Medical 3D printing with polyjet technology: Effect of material type and printing orientation on printability and cytotoxicity

Karl H. Schneider, Gunpreet Oberoi, Ewald Unger, Klara Janjic, Sabrina Rohringer, Stefan Heber, Hermann Agis, Andreas Schedle, Herbert Kiss, Bruno K. Podesser, Stefan Toegel, and Francesco Moscato

Statistical Analysis

Dimensions

Two separate 2-way analyses of variance were performed to investigate whether the precision in height or diameter, defined as the absolute deviation from the target value (height 2 mm, diameter 5 mm) differs between materials and whether this in turn is affected by printing direction. Significant 'material*direction' interactions were broken down using pairwise comparisons of the absolute deviations between the two printing directions within each material. These contrasts were based on Bias-corrected and accelerated (BCa) bootstraps and thus can be considered robust.

Roughness

The two parameters reflecting roughness (mean peak, Ra, and averages roughness, Rz) were analyzed separately by mixed linear models. There were 4 specimens per material (VC, VPW, M610) per printing direction (horizontal, vertical), i.e. 24 in total. Each specimen was assessed three times for Ra and three times for Rz, i.e. 144 data points were available in total, 72 referring to Ra and 72 to Rz. To account for the fact that three measurements were taken for each specimen, each specimen was used as level of a random factor. Significant 'material*direction' interaction terms were broken down using contrasts comparing the two printing directions within each material.

Toxdent

To test whether materials are cytotoxic as measured by the Toxdent-Assay, a linear mixed model was used. Samples were prepared in 6-well plates, of which in total 60 were used, corresponding to 360 data points. Experiments were performed in two experimental runs of 30 plates each. Within each experimental run, 15 plates were used for materials printed in horizontal direction, and 15 for materials printed in vertical direction (or the corresponding controls). Plates were randomly assigned to each of the 5 experimental conditions/materials (neg CTR, pos CTR, VC, VPW, M610), i.e., each plate contained only wells with a certain material in a certain printing direction. This resulted in 3 plates per material per printing direction. To allow an intuitive interpretation of the results (i.e., in % viability), data were normalized to the mean of the neg CTR values, separately by experimental run.

To account for the correlation of data within each run and each plate, the variable ‘run’ with the levels “first” and “second” and variable ‘plate’ with each of the 60 plates as levels were included as random factors in the model.
The printing direction was used as binary fixed factor, and the materials were represented as fixed factor with four levels, since the neg CTR was omitted from the analysis as it was used for normalization. To test the possibility that printing direction might affect some, but not other materials, a ‘material*direction’ interaction was tested first. As this was not significant, the interaction term was dropped from the model. The least square means and P-values are derived from the model without the interaction. Prior to analysis, the data were log_{10}-transformed, therefore, least square means are geometric means. Each of the least-square means was tested against 70% to assess whether the geometric mean was different from the *a priori*-defined threshold for toxicity.

MTT

The research question regarding the MTT assay was whether the toxicity of materials depends on printing direction and whether this in turn is dependent on the laboratory, corresponding to a three-way interaction hypothesis. The factors were 1) ‘material’ (5 levels: i. cement/pos CTR, ii. VC, iii. VPW, iv. M610), 2) ‘printing direction’ with the levels “vertical”, “horizontal” and “not printed”, whereby the materials “glass” and “cement” were always not printed and 3) ‘laboratory’ with the levels “CBMR”, “DENT” and “OTS”.
The laboratories CBMR and DENT always used 60 wells per 96-well plate for acquisition of the absorbance measurements at 570 nm, 48-wells were used in the OTS laboratory. For each biological replicate (defined as cells exposed to a specimen), the solution was transferred to four (CBMR, DENT) or two (OTS) wells of the measurement plates, generating technical replicates. In the laboratories CBMR and DENT, 15 biological samples were analyzed per plate, resulting in 60 wells per plate. In the OTS laboratory, 24 biological replicates were analyzed per plate, resulting in 48 wells. On each plate of every laboratory, 3 biological replicates of glass serving as negative control were analyzed, allowing to normalize the other values on this plate to the negative control. Normalization was done for each plate separately by dividing the measured value of a well by the arithmetic mean of all wells corresponding to the glass exposure. The normalized values were used as dependent variable.
For statistical analysis, a linear mixed model was applied, with included the three above-mentioned factors as fixed factors. Each biological replicate was represented by a level of the random factor ‘replicate’, which was used to account for the dependency of technical replicates originating from a single biological replicate. Furthermore, each plate was represented by a level of a random factor ‘plate’ to account for the dependency of measured values being measured simultaneously on a single plate. All values corresponding to “glass” were not included in the statistical model, as they were used for normalization.

First, a full-factorial model was fit with all three 2-way interaction terms and the 3-way interaction, i.e. the interaction ‘laboratory*material*printing direction’. The latter was used to test the hypothesis that a possible print-direction specific effect was different between laboratories. As this was non-significant, it was omitted from the model. Next, the non-significant two-way interaction ‘laboratory*material’ was dropped, which corresponds to the hypothesis that the effects of different materials on viability is laboratory specific. From the resulting model, least square means were calculated for each condition with their 95% confidence intervals and tested against the *a priori*-defined cut-off for toxicity of 70% viability.

MTT primary cells

Primary cells were exposed to the materials to test potential toxicity in two laboratories. The CBMR laboratory analyzed HUVECs from 5 different donors, whereas the OTS analyzed both chondrocytes and fibroblast-like synoviocytes (FLS) from three donors, whereby each of the three patients donated both chondrocytes and FLS. Cells of each donor were exposed to glass, cement, M610, VC, VPW, the latter three of which in both printing directions, i.e., 8 conditions. At CBMR, each of these conditions, were carried out thrice per donor, corresponding to 24 wells per donor. At OTS, each condition was performed in six times, corresponding to 48 wells per donor. Measured absorbance from the wells were first normalized for each plate separately by dividing the measured value of a well by the arithmetic mean of all wells corresponding to the glass exposure within that plate. The normalized values were used as dependent variable.

The analysis was performed by a linear mixed model. The fixed factors were 1) ‘material’ (4 levels i. cement/pos CTR, ii. VC, iii. VPW, iv. M610), 2) ‘printing direction’ with the levels “vertical”, “horizontal” and “not printed”, whereby “cement” was always not printed and 3) ‘cell type with the levels “HUVECs”, “chondrocytes” and “FLS”. The donors were included as levels of a random factor. To stabilize the residual distribution, the normalized values were $\log_{10}$ transformed prior to analysis. First, a full factorial model was fitted. Following this, the three-way interaction term was omitted, thereafter non-significant two-way interactions were removed. The estimated means (of $\log_{10}$ transformed values) for each experimental group of interest were tested against 1.8451, i.e. against 70% on the original scale, to assess whether the percentage viability is different from this value.

High Resolution imaging

One-shot high-resolution 3D macroscopic measurements with a resolution of 0.1 µm were performed using a VR-5200 camera system (Keyence, Keyence International, Mechelen, Belgium). This device acquires measurement data at 800,000 points in four seconds with a working distance of 75 mm and offers a resolution of 0.1 µm.

1.1 VC vertical

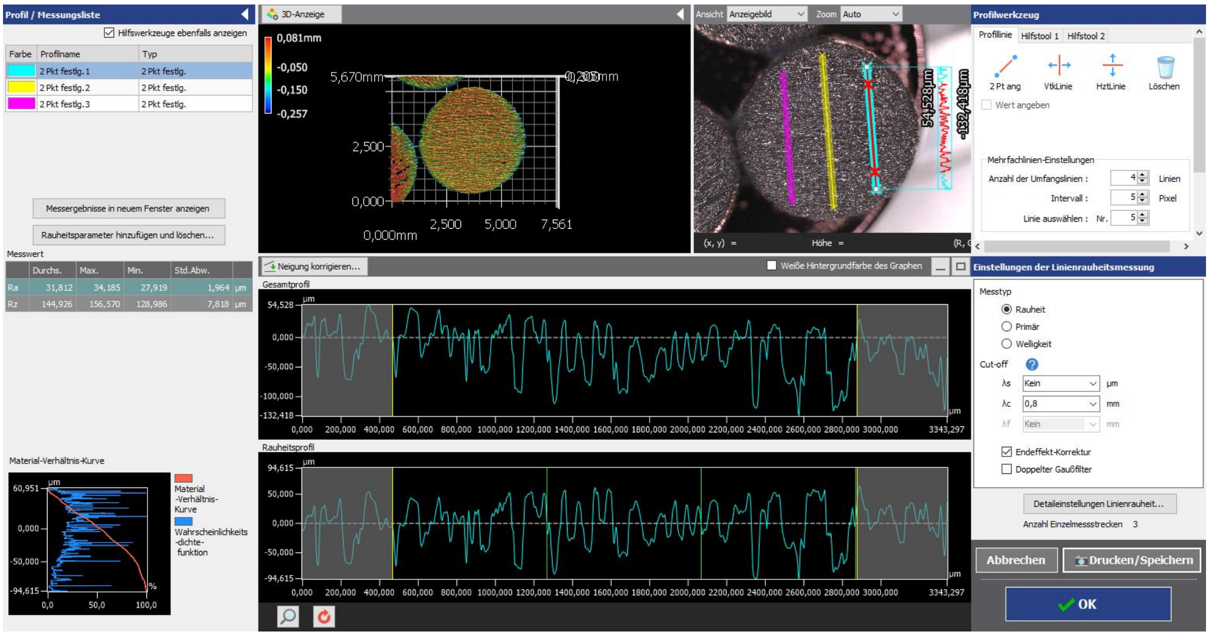

1.2 VC horizontal

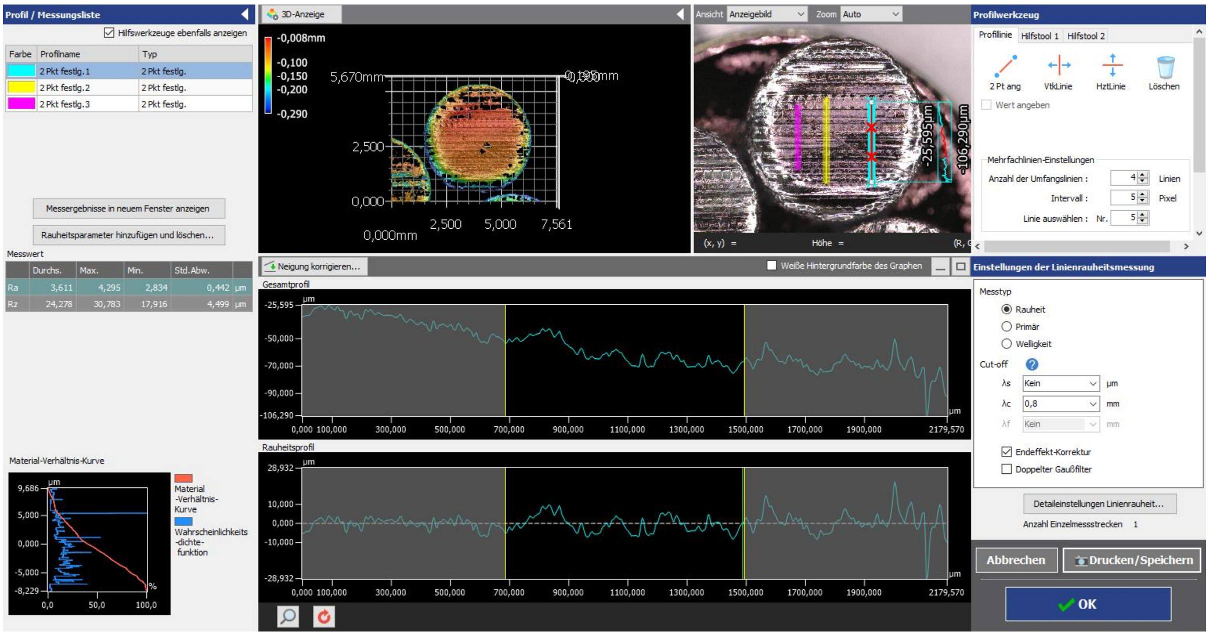

1.3 VPW vertical

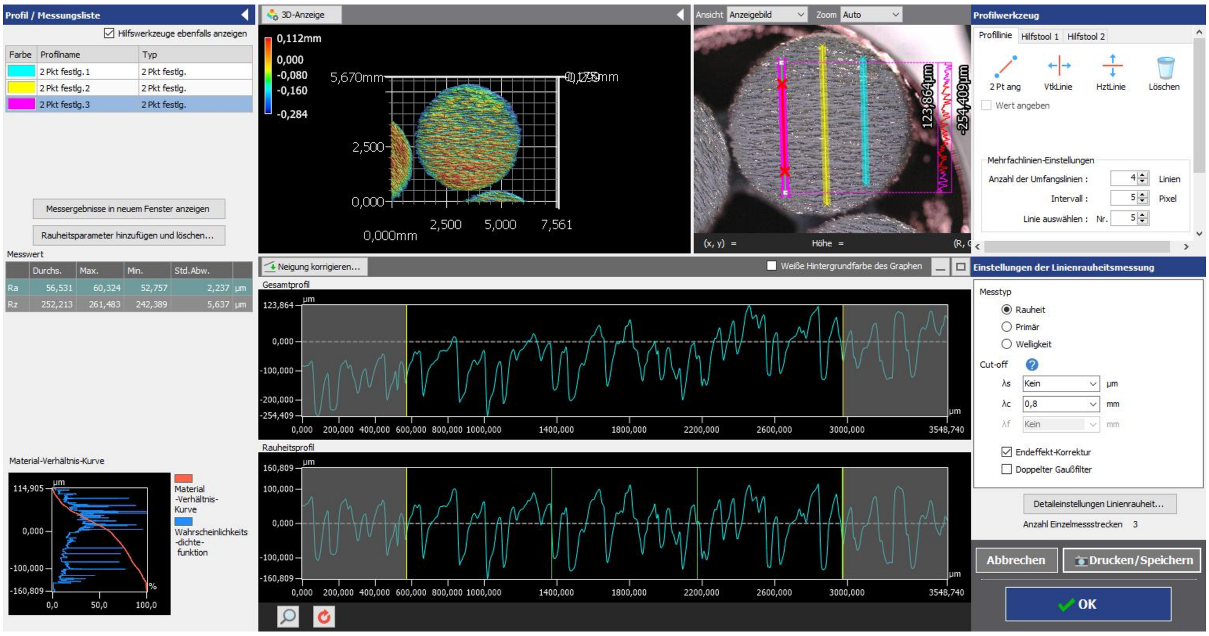

1.4 VPW horizontal

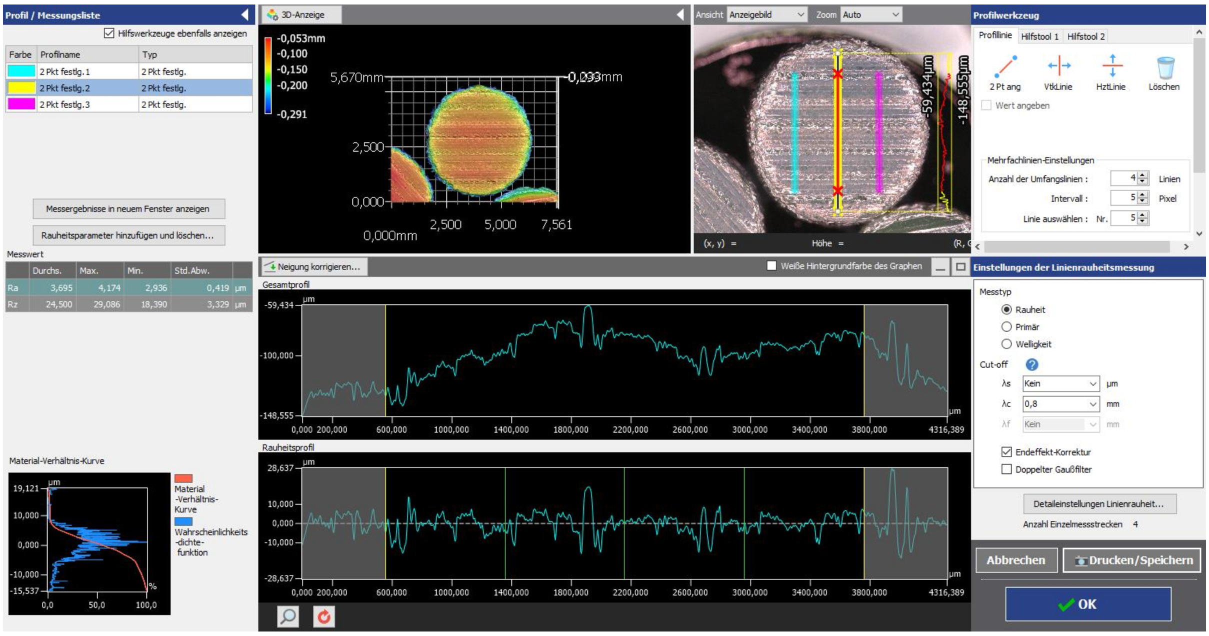

1.5 M610 vertical

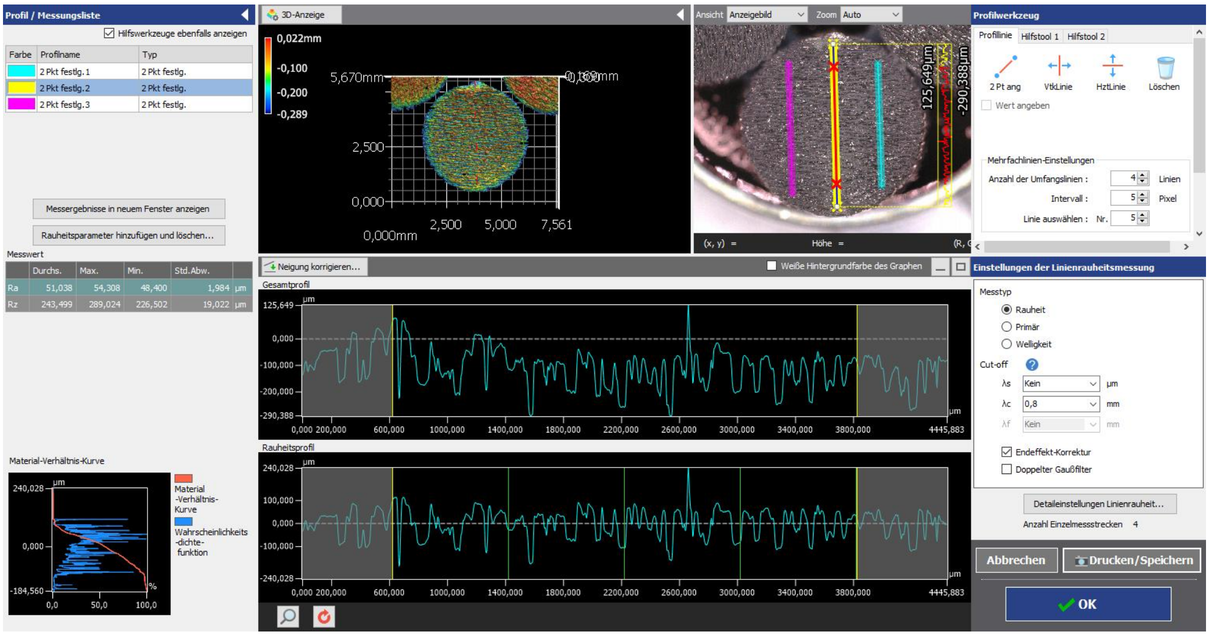

2.6 M610 horizontal:

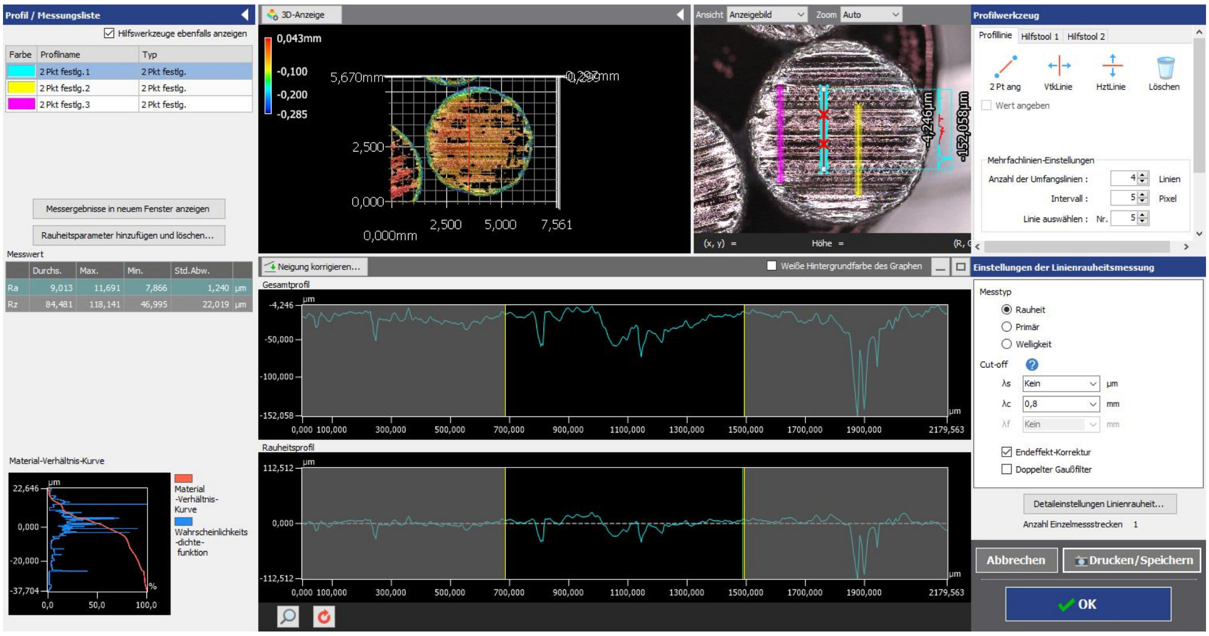

Roughness and radius determined by high resolution imaging

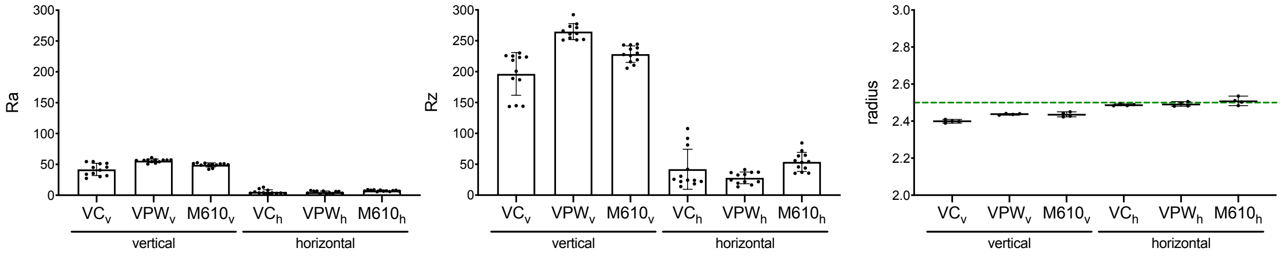

Dimensional measurements: raw data

Discs dimensions were determined from 50 samples of each group using a Mitutoyo 7309 Pocket Thickness Gage (0-10mm Range, 0.01mm Graduation, 20 micrometer accuracy).

	Height	Diameter	Height	Diameter	Height	Diameter	Height	Diameter	Height	Diameter	Height	Diameter
	Horizontal	Horizontal	Horizontal	Horizontal	Horizontal	Horizontal	Vertical	Vertical	Vertical	Vertical	Vertical	Vertical
	VC	VC	VPW	VPW	Med610	Med610	VC	VC	VPW	VPW	Med610	Med610
**1**	2,02	5,01	2,07	4,94	2,01	5,17	2,1	5,06	2,16	4,98	2,16	4,96
**2**	2,01	5,01	2,06	5,13	2	5,11	2,07	4,96	2,16	4,94	2,17	4,99
**3**	2,04	4,91	2,1	5,13	2,02	5,09	2,13	4,88	2,16	5,02	2,14	4,97
**4**	2,02	4,9	2,05	4,99	2,01	5,16	2,1	4,8	2,15	5,02	2,19	5,02
**5**	2,01	5,03	2,05	5,11	2	5	2,11	5,02	2,14	4,95	2,15	5,06
**6**	2,02	4,97	2,05	5,06	2,02	5,12	2,15	4,77	2,16	4,96	2,17	4,94
**7**	2,05	5,04	2,08	5,06	2,01	5,14	2,13	5,03	2,12	4,95	2,15	4,98
**8**	2,09	4,93	2,04	5,15	2	5,1	2,06	4,96	2,14	4,97	2,15	4,98
**9**	2,02	5,04	2,07	5,14	2	5,01	2,15	4,88	2,16	5,94	2,21	4,88
**10**	2	5	2,08	5,06	2,01	5,09	2,11	4,97	2,17	4,97	2,2	4,98
**11**	2,01	4,93	2,07	5,11	2,11	5,1	2,15	4,85	2,16	5,06	2,18	4,95
**12**	2,05	5,05	2,06	4,92	2,02	5,13	2,09	4,95	2,15	4,98	2,17	4,95
**13**	2,02	5,01	2,07	5,1	2,02	4,97	2,08	4,96	2,14	5,04	2,15	4,92
**14**	2,04	5,11	2,04	5,12	2,01	5,05	2,1	4,96	2,15	4,96	2,15	4,98
**15**	2,02	5,01	2,08	5,15	2,11	5	2,09	4,85	2,13	5,04	2,19	4,95
**16**	2,08	5,03	2,05	5,09	2,01	5,07	2,1	4,93	2,17	4,98	2,19	5,03
**17**	2,04	4,96	2,09	4,99	2,14	5,16	2,1	4,85	2,17	4,99	2,14	5,08
**18**	2,03	5,04	2,08	4,97	2	5,11	2,11	4,92	2,15	5,05	2,12	4,98
**19**	2,05	4,94	2,07	5,11	2	4,97	2,15	4,77	2,16	5,07	2,18	4,99
**20**	2,01	5,01	2,1	4,94	2,02	5,05	2,14	4,94	2,15	5,05	2,15	5,02
**21**	2,02	4,96	2,07	5,06	2,02	5,05	2,13	4,77	2,13	5,96	2,16	5,04
**22**	2,01	5,04	2,1	5,07	2	5,1	2,13	5,04	2,1	4,94	2,18	4,95
**23**	2,02	4,91	2,07	5	2	5,07	2,08	4,9	2,15	4,95	2,17	5,07
**24**	2	5,02	2,1	4,96	2,01	5,16	2,11	4,92	2,13	4,97	2,19	4,95
**25**	2,01	5,03	2,03	5,09	2,02	5,09	2,14	5,01	2,17	5,05	2,13	5,02
**26**	2,03	5	2,03	5,11	2,11	4,96	2,1	4,96	2,13	5	2,16	4,94
**27**	2,09	4,99	2,05	5,15	2,04	5,03	2,1	4,89	2,16	4,94	2,19	4,87
**28**	2,01	5,1	2,1	4,96	2	5,09	2,12	4,96	2,17	4,94	2,21	5,06
**29**	2,09	5,1	2,05	5,05	2,01	5,06	2,13	5	2,13	5,07	2,16	4,95
**30**	2,02	5,09	2,05	5,03	2,04	5,12	2,09	4,83	2,14	5,1	2,17	4,99
**31**	2,01	5,03	2,09	4,99	2	5,09	2,1	4,77	2,13	5,02	2,17	4,89
**32**	2,04	4,97	2,1	5,16	2,01	5,09	2,12	4,83	2,16	4,97	2,15	5,04
**33**	2,01	5,12	2,12	5,13	2,01	4,98	2,12	5,03	2,15	4,92	2,14	4,95
**34**	2,08	5,12	2,07	5,06	2,02	5,12	2,14	4,92	2,16	4,99	2,18	5,01
**35**	2,01	4,96	2,06	5,16	2,11	5	2,12	4,98	2,11	4,93	2,15	4,98
**36**	2	5,06	2,09	5,01	2,04	4,96	2,13	4,76	2,15	5,03	2,17	4,95
**37**	2,02	4,91	2,04	5,11	2,01	5,17	2,13	4,88	2,13	4,99	2,16	5
**38**	2,01	5,01	2,05	4,96	2,01	5	2,1	4,82	2,13	4,88	2,16	4,94
**39**	2,05	5,06	2,09	5,12	2,01	5,06	2,08	4,96	2,13	5,16	2,17	4,96
**40**	2,02	5,03	2,09	5,12	2,02	5,07	2,1	4,9	2,15	4,97	2,18	4,98
**41**	2,03	5,03	2,1	4,97	2,02	5,16	2,1	4,83	2,12	4,91	2,16	4,98
**42**	2,03	5,12	2,11	5,12	2,01	5,09	2,12	4,88	2,14	4,94	2,15	4,94
**43**	2,01	5,01	2,09	5,05	2	5,01	2,09	5	2,15	5,02	2,15	4,94
**44**	2,02	4,94	2,07	5,1	2,02	5,11	2,08	4,8	2,16	4,99	2,17	5,05
**45**	2,01	4,93	2,07	4,98	2,03	4,97	2,1	4,91	2,15	4,92	2,16	4,85
**46**	2,05	4,93	2,08	5,13	2,01	5,09	2,11	4,88	2,16	5,03	2,17	4,96
**47**	2,02	4,98	2,04	5,12	2	5,03	2,12	4,83	2,17	4,91	2,2	5,07
**48**	2,03	4,92	2,08	5,11	2,02	4,96	2,1	4,83	2,16	5,03	2,15	4,95
**49**	2,05	5	2,09	5	2,02	4,93	2,13	4,78	2,15	4,96	2,11	4,9
**50**	2,09	5,11	2,1	5,12	1,99	4,99	2,12	4,91	2,15	4,95	2,18	
**Mean**	**2,03**	**5,01**	**2,07**	**5,07**	**2,02**	**5,06**	**2,11**	**4,90**	**2,15**	**5,03**	**2,17**	**4,98**
**StDev**	**0,03**	**0,06**	**0,02**	**0,07**	**0,03**	**0,07**	**0,02**	**0,08**	**0,02**	**0,20**	**0,02**	**0,05**
